# Molecular van der Waals Fluids in Cavity Quantum Electrodynamics

**DOI:** 10.1021/acs.jpclett.3c01790

**Published:** 2023-09-29

**Authors:** John P. Philbin, Tor S. Haugland, Tushar K. Ghosh, Enrico Ronca, Ming Chen, Prineha Narang, Henrik Koch

**Affiliations:** † Harvard John A. Paulson School of Engineering and Applied Sciences, Harvard University, Cambridge, Massachusetts 02138, United States; ‡ College of Letters and Science, University of California, Los Angeles, California 90095, United States; § Department of Chemistry, Norwegian University of Science and Technology, 7491 Trondheim, Norway; ∥ Department of Chemistry, Purdue University, West Lafayette, Indiana 47907, United States; ⊥ Dipartimento di Chimica, Biologia e Biotecnologie, Università degli Studi di Perugia, Via Elce di Sotto, 8, 06123 Perugia, Italy; # Max Planck Institute for the Structure and Dynamics of Matter and Center Free-Electron Laser Science, Luruper Chaussee 149, 22761 Hamburg, Germany; ¶ College of Letters and Science, University of California, Los Angeles, California 90095, United States; ∇ Scuola Normale Superiore, Piazza dei Cavalieri, 7, 56124 Pisa, Italy

## Abstract

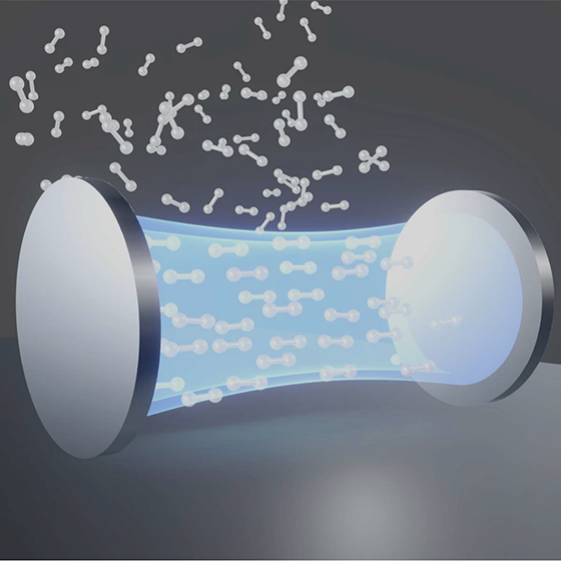

Intermolecular van der Waals interactions are central to chemical and physical phenomena ranging from biomolecule binding to soft-matter phase transitions. In this work, we demonstrate that strong light–matter coupling can be used to control the thermodynamic properties of many-molecule systems. Our analyses reveal orientation dependent single molecule energies and interaction energies for van der Waals molecules. For example, we find intermolecular interactions that depend on the distance between the molecules *R* as *R*^–3^ and *R*^0^. Moreover, we employ *ab initio* cavity quantum electrodynamics calculations to develop machine-learning-based interaction potentials for molecules inside optical cavities. By simulating systems ranging from 12 H_2_ to 144 H_2_ molecules, we observe varying degrees of orientational order because of cavity-modified interactions, and we explain how quantum nuclear effects, light–matter coupling strengths, number of cavity modes, molecular anisotropies, and system size all impact the extent of orientational order.

van der Waals interactions are ubiquitous in chemistry and physics, playing important roles in diverse scientific fields ranging from DNA base stacking to 2D material interlayer interactions.^1−3^ There has been a long history of attempting to elucidate the origin of van der Waals interactions;^4,5^ the first quantum mechanical derivation was performed by London in the 1930s using second-order perturbation theory.^6^ London found that two molecules that do not have permanent dipoles (e.g., H_2_), which we refer to as van der Waals molecules, have an attractive interaction between them that scales with the distance between the molecules *R* as *R*^–6^.^6^ This *R*^–6^ attractive force is commonly used as the long-distance asymptotic form of van der Waals interactions in many force fields and to correct van der Waals interactions in *ab initio* calculations, which have both achieved great successes in modeling thermodynamic properties in a variety of systems.^7,8^ Despite van der Waals interactions being central to many properties of molecular and condensed matter systems, limited approaches have been proposed to manipulate intermolecular van der Waals interactions. However, applied electromagnetic fields have been shown to modify van der Waals interactions between atoms and molecules,^9−12^ and Haugland et al.^13^ recently showed numerically that van der Waals interactions are significantly altered by strong light–matter coupling in optical cavities. These studies open the possibility of controlling the properties and structure of molecular fluids by tuning the light–matter coupling parameters, the coupling strength, and frequency.

The goal of this work is to understand how the structure of molecular van der Waals fluids can be modulated using enhanced vacuum electromagnetic quantum fields, and we focus on the impact that a single strongly coupled photon mode can have on the properties of model molecular van der Waals fluids. To this end, we leverage recent developments in cavity quantum electrodynamics (QED) simulations and neural network pair potentials to simulate molecular fluids of H_2_ molecules strongly coupled to a single photon mode (Figure 1). By analyzing how cavity-modified single molecule energies and cavity-mediated intermolecular interactions depend on the orientation of the H_2_ molecules both relative to the cavity polarization vector and relative to one another, we can explain how cavities impact the structure and orientational order of molecular van der Waals fluids. The findings reported herein should readily be transferable to other molecules and light–matter regimes (e.g., vibrational polaritons) given the generality of the cavity QED Hamiltonian used in this work.^14−19^ We also discuss how the light–matter coupling strength, number of cavity modes, anisotropic polarizabilities of molecules, quantum nuclear effects, and molecular concentrations can all impact the extent of orientational order observed in any particular cavity QED experiment.^20−24^

**Figure 1:**
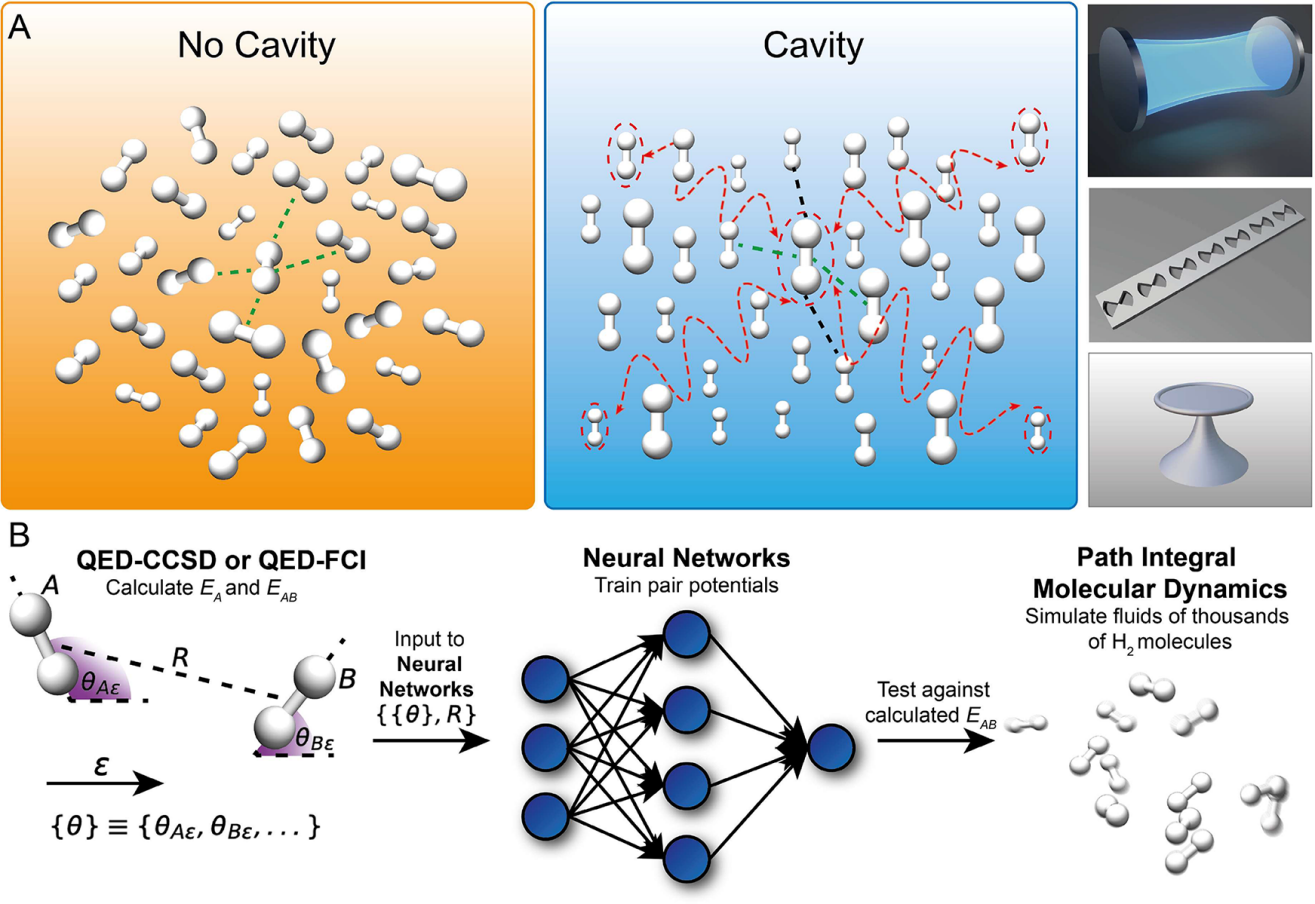
(A) Schematic representation of the findings from our simulations of a fluid of H_2_ molecules outside and inside a cavity. Specifically, orientational order can be observed inside a cavity, whereas the H_2_ molecules can rotate freely outside a cavity. The dashed lines represent the different intermolecular interaction length scales outside and inside a cavity. (B) Diagram describing the computational workflow used in this work. *Ab initio* cavity QED energies and corresponding symmetry preserving features (see Figure S3, Table S3 and section IV.A.1 for details of symmetry preserving features) of many 2H_2_ configurations are used to develop neural network-based intermolecular pair potentials capable of being utilized in path integral molecular dynamics simulations of fluids of H_2_ molecules.

In molecular dynamics (MD) simulations, the nuclei move along electronic potential energy surfaces. In the cavity case, where the photon contributions are added, these surfaces have been termed polaritonic potential energy surfaces.^25−27^ In both cases, the total potential energy of *N* H_2_ molecules can be calculated as a many-body expansion,
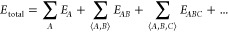
1where *E*_*A*_ represents the single-molecule energies, *E*_*AB*_ represents the intermolecular interaction energies between all unique pairs of molecules, and so on for higher-body terms. In this work, we focus on contributions to the total energy in eq 1 arising from at most two-body interactions. The three-body and higher-body terms are significantly smaller than the two-body interactions per interaction; see the Supporting Information for details. Outside the cavity, the one-body term does not depend on the orientation of the H_2_ molecule. On the other hand, inside the cavity, the molecule–field interaction causes the one-body energies to depend on the orientation of the H_2_ molecules with respect to the optical cavity polarization vector, **ε**.^28^ Furthermore, the two-body energies depends on the orientation between the two molecules as well as their orientation relative to the field as a consequence of the anisotropic polarizability of H_2_ molecules, in contrast to isotropic polarizabilities of atoms.^9−12,29^

We calculate *E*_*A*_ and *E*_*AB*_ by solving the Schrödinger equation for the cavity QED Hamiltonian in the dipole approximation with a single photon mode using accurate coupled cluster (QED-CCSD-12-SD1) and near exact full configuration interaction (QED-FCI-5).^30^ Our single photon mode has a coupling constant of λ = 0.1 au and energy of ℏω_*c*_ = 13.6 eV unless specified otherwise. This coupling constant is rather large as it corresponds to the coupling of at least 5 independent modes, where each has an effective volume of 0.9 nm^3^. Modeling multiple modes by a single effective mode with a larger coupling constant is exact for mean-field cavity QED methods but is an approximation otherwise. We detail below how the cavity-modified local interactions and cavity-induced collective effects depend on λ. More than 100 000 H_2_ dimer configurations are used as inputs to a fully connected neural network that serves as our intermolecular pair potential, which is trained and tested against the calculated energies. The trained potential energy functions were carefully tested, and in the Supporting Information, we demonstrate that our machine learning models are fully capable of reproducing the potential energy surfaces. In Figure 1B, we show the computational workflow used in this work schematically. In this study, we focus on path integral molecular dynamics (PIMD) simulations in order to account for quantum nuclear effects. Our PIMD simulations of fluids of H_2_ molecules were performed with a fixed number of molecules (*N*), temperature (*T*), and volume (*V*). All PIMD simulations presented herein were performed with a molecular density of 13 molecules per nm^3^, a temperature of 70 K, and *N* = 12 unless otherwise specified. More details on the simulations, including comparisons of QED-CCSD-12-SD1 with QED-FCI-5, comparisons of MD with PIMD, and additional parameter regimes (e.g., smaller λ values), are provided in the Supporting Information.

The structural properties of the molecular van der Waals fluids are analyzed using PIMD simulation trajectories. In Figure 2, we summarize the main findings of our PIMD and classical MD simulations. Figure 2A and Figure 2B show representative thermal equilibrium configurations for the no cavity (orange) and cavity (blue) scenarios, respectively. The impact of the cavity-modified interactions is observable in the orientational order of the H_2_ molecules both relative to the cavity polarization vector (θ_*Aε*_, Figure 2C,E,F) and relative to other H_2_ molecules (θ_*AB*_, Figure 2D). Specifically, Figure 2C–F shows that the cavity-modified energies enhance the probability of finding two molecules oriented parallel to one another (i.e., θ_*AB*_ = 0, π) and perpendicular to the cavity polarization vector (i.e., θ_*A*ε_ = ^π^/_2_). However, the extent of this orientational order depends on many factors, including the magnitude of quantum nuclear effects, the light–matter coupling strengths, molecular anisotropies, and number of molecules. To elucidate the importance of quantum nuclear effects, we compare the orientational order observed in PIMD simulations of H_2_, D_2_, and T_2_ with a classical MD simulation of H_2_ in Figure 2C; the degree of orientational order monotonically increases upon increasing the molecular masses from H_2_ to D_2_ to T_2_ (which reduces quantum nuclear effects) and is further enhanced when quantum nuclear effects are completely removed as in the classical MD simulation. Next, in Figure 2D–F, we show how cavity-modified one-body energies and two-body intermolecular energies each impact the orientational order. Figure 2D and Figure 2E demonstrate that the cavity-modified one-body energies are the dominant driver of the orientational order for the case of 12 H_2_ molecules. The orange lines in Figure 2D,E show that the H_2_ molecules have no preferred orientation axis outside the cavity, consistent with the global rotational symmetry of the electronic and nuclear Hamiltonian in the absence of the cavity. However, the presence of the bilinear coupling and dipole self-energy terms breaks this symmetry such that H_2_ molecules prefer to orient their bond axis in specific orientations relative to the cavity polarization vector and relative to one another. In particular, the dipole self-energy term outcompetes the bilinear coupling term and is responsible for the 12 molecule simulations preferentially aligning perpendicular to the cavity polarization vector (Figure 3A). However, Figure 2E,F demonstrates that the cavity-modified one-body energies lead to this perpendicular alignment, whereas the cavity-modified two-body intermolecular interactions attempt to align the molecules parallel to the cavity polarization vector. Specifically, the green line in Figure 2E shows that the cavity-modified one-body term causes H_2_ molecules to preferentially align perpendicular to the cavity polarization vector (i.e., θ_*A*ε_ = ^π^/_2_), and the inclusion of cavity-modified two-body interactions begins to counteract this effect as seen in the blue line in Figure 2E reducing the orientational alignment. This effect of the two-body interactions causing the H_2_ molecules to preferentially align parallel to the cavity polarization vector (i.e., θ_*A*ε_ = 0, π) and the collective nature of the cavity-modified intermolecular interactions are highlighted in Figure 2F and Figure S13. We find that for a small number of molecules (e.g., *N* = 12) the one-body term dominates and the molecules preferentially align perpendicular to the cavity polarization vector, but as *N* increases to 144 H_2_ molecules with a fixed coupling and cavity volume, the orientational order is lost due the cavity-modified one-body and two-body effects perfectly canceling one another. On the other hand, the 12 and 144 H_2_ molecule simulations excluding the cavity-modified van der Waals interactions overlay one another (green lines in Figure 2F). Additionally, the extent of orientational order induced by the cavity decreases as the light–matter coupling strength decreases as shown in Figure S8 and explained analytically below.

**Figure 2:**
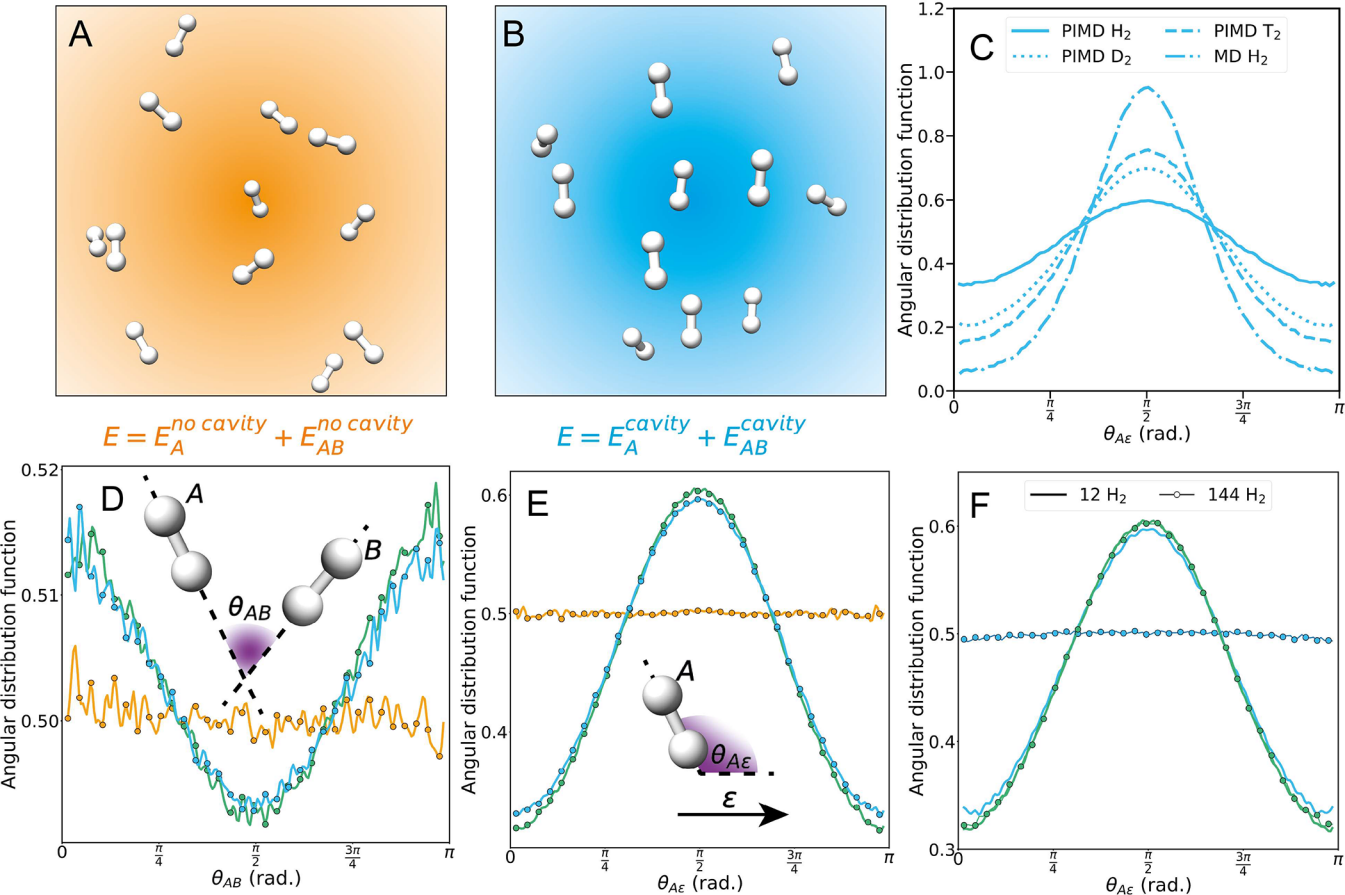
(A, B) Snapshots taken at thermal equilibrium from molecular dynamic (MD) simulations in the cases of (A) no cavity (orange) and (B) cavity-modified one-body and two-body terms (blue). (C) The impact of quantum nuclear effects is demonstrated by comparing the molecular bond axis to cavity polarization vector (θ_*A*ε_), angular probability distribution function *P*(θ_*A*ε_) for path integral molecular dynamics (PIMD) simulations of H_2_, D_2_, T_2_, and a classical MD simulation of H_2_. (D) Molecular bond axis of molecule *A* to molecular bond axis of molecule *B* (θ_*AB*_) angular probability distribution function *P*(θ_*AB*_) and (E) *P*(θ_*A*ε_) are shown for PIMD simulations for no cavity (orange), cavity (blue), and cavity-modified one-body term but no cavity two-body term (green) cases. (F) *P*(θ_*A*ε_) is shown for PIMD simulations containing different numbers of H_2_ molecules within the same cavity volume (i.e., changing the molecular density) for cavity (blue) and cavity-modified one-body term but no cavity two-body term (green) cases. All PIMD simulations shown in this figure were performed using neural networks trained with CCSD (no cavity) or QED-CCSD-12-SD1 with λ = 0.1 au (cavity) calculated energies. All entropic contributions to angle distribution functions are removed.

**Figure 3:**
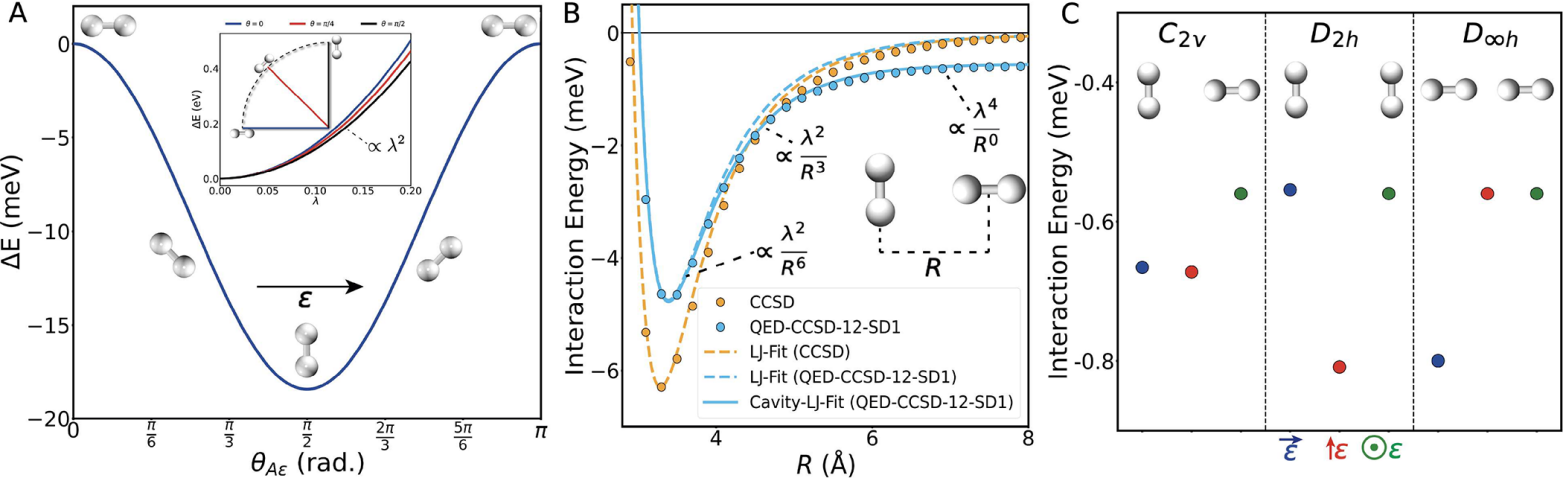
(A) Energy difference, Δ*E*, between a single H_2_ molecule inside a cavity aligned perfectly along the cavity polarization vector, **ε**, and different angles relative to the cavity polarization vector. The inset shows that the energy of a single molecule within a cavity increases with λ^2^. (B) Intermolecular interaction energies, *E*_*AB*_, and fits to a Lennard-Jones type potential given by eq 3 (dashed lines) and cavity-modified Lennard-Jones type potential given by eq 4 (solid line). (C) Intermolecular interaction energies, *E*_*AB*_, at 25 Å for various high-symmetry molecular orientations and cavity polarizations. All calculations shown in this figure were performed using QED-CCSD-12-SD1 with λ = 0.1 au.

Although we performed nonperturbative *ab initio* cavity QED calculations, perturbation theory can be used to further analyze and explain the major findings of our PIMD and MD simulations. We summarize our key findings here and in Figure 3, and the complete analysis is provided in the Supporting Information. The cavity modifications to the one-body energies, *E*_*A*_, results in the H_2_ molecules aligning their bonds orthogonal to the cavity polarization. This occurs because H_2_ is most polarizable along its bond axis, and from perturbation theory, we can obtain an expression for the cavity-modified one-body energy as
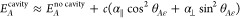
2where α_∥_ and α_⊥_ are the polarizabilities of molecular hydrogen along its bond axis and perpendicular axes, respectively, and *c* is a positive scalar constant proportional to the molecule–cavity coupling squared (i.e., *c* ∝ λ^2^). Equation 2 is in agreement with the *ab initio* calculations shown in Figure 3A. Interestingly, the dipole self-energy term increases the energy of a single molecule in a cavity more than the bilinear coupling term decreases the energy (eq S12); thus, the lowest energy orientation of a single molecule in a cavity is such that its most polarizable axis is perpendicular to the cavity polarization vector (or vectors in terms of multimode cavities).

In terms of the cavity modifications to the two-body energies, Figure 3B shows the intermolecular interaction between two H_2_ molecules as a function of the center-to-center distance (*R*). The impact of the cavity on this dissociation curve at first glance appears modest, even for the rather large light–matter coupling of λ = 0.1 au, but these modifications can impact the structural and thermodynamic properties of molecular van der Waals systems for a few reasons. First, a standard intermolecular van der Waals interaction potential given by
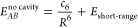
3where *E*_short-range_ accounts for the short-range repulsion between van der Waals molecules and the *R*^–6^ term is the usual attractive London dispersion interaction, is not applicable inside an optical cavity (Figure 3B).^9−12^ A modified interaction potential that includes angle-dependent terms that scale as *R*^–3^ and *R*^0^ is necessary inside an optical cavity such that the interaction between two van der Waals molecules is given by
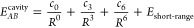
4These cavity-induced interactions between van der Waals molecules arise as early as second-order perturbation theory (see Supporting Informationeq S9).^9^ The *R*^0^ interaction between a single pair of molecules is rather weak (*c*_0_ ∝ λ^4^) as shown in Figure 3C. However, due to its long-range nature, a single molecule interacts with all other molecules, and thus, the collective effect of this interaction can become large in many-molecule simulations. Importantly, this interaction strength depends on the orientations of both molecular bonds relative to the cavity polarization (Figure 3C). Specifically, the interaction energy is minimized when the molecular bonds of both molecules are parallel to the cavity polarization vector because the interaction strength of this term is approximately related to the product of the polarizability of each molecule along **ε** (*c*_0_ ∝ α_*A*ε_α_*B*ε_). And because *c*_0_ is always negative, this *R*^0^ intermolecular interaction increases the probability of finding H_2_ molecules parallel to the cavity polarization vector and decreases the probability of finding the molecules perpendicular to the polarization vector (Figure 2E,F). The collective nature of this interaction is demonstrated in Figure 2F and Figure S13 where the orientational order depends on the number of H_2_ molecules for simulations with the same simulation volume but different molecular densities. At *N* = 144, the orientational order due to the two-body interactions has become so large that they entirely cancel out the orientational effects from the cavity modified one-body energies that are dominated by dipole self-energy effects for *N* = 12 molecules. As *N* increases further, we expect that the system will completely flip and instead align parallel to the polarization vector. This is demonstrated in the Supporting Information (Figure S13), but the number of molecules required (*N* ≥ 1000) is too large to justify in a realistic system with the coupling we are using currently. Both the cavity-modified *R*^–6^ and cavity-induced *R*^–3^ interactions scale with λ^2^ at the lowest order. Importantly, the *R*^–3^ interaction is not a result of the cavity inducing a dipole moment in the H_2_ molecules but rather an interaction taking place via the cavity mode. As discussed in the Supporting Information in more detail, the intermolecular angle and molecule–cavity angle dependencies of the perturbation potential combine to create the orientational order shown throughout Figure 2.

In summary, we have demonstrated that strong light–matter coupling to a single photon mode can have profound impacts on the properties of molecular van der Waals fluids by combining *ab initio* cavity QED calculations with path integral molecular dynamics simulations of many H_2_ molecules. We found that cavity-modified single molecule and intermolecular interaction energies result in significantly changed molecular orientational order, even in the fluid phase. We look forward to seeing future experimental and theoretical studies that aim to elucidate how processes such as ion and molecular diffusion, intermolecular energy transfer,^31−33^ and chemical reactivity^16,34−38^ are impacted by the unique properties of molecular fluids in cavity QED reported here.

## Supporting Information Available

Additional details on our *ab initio* calculations, perturbation theory derivations, and molecular dynamics simulations (PDF)
