# Supplementary material for: Molecular van der Waals Fluids in Cavity Quantum Electrodynamics
Source: J Phys Chem Lett. 2023 Sep 29;14(40):8988–93. doi: 10.1021/acs.jpclett.3c01790 (PMC10578074; doi:10.1021/acs.jpclett.3c01790)
Supplement: Supplementary file 1 — jz3c01790_si_001.pdf [file jz3c01790_si_001.pdf]

Supplementary Information: Molecular van der Waals Fluids in Cavity Quantum Electrodynamics

John P. Philbin¶,[1,2,*] Tor S. Haugland¶,[3] Tushar K. Ghosh¶,[4] Enrico Ronca,[5,6] Ming Chen,[4,†] Prineha Narang,[1,2,‡] and Henrik Koch[3,7,§]

[1] *Harvard John A. Paulson School of Engineering and Applied Sciences, Harvard University, Cambridge, MA 02138, USA*
[2] *College of Letters and Science, University of California, Los Angeles, CA 90095, USA*
[3] *Department of Chemistry, Norwegian University of Science and Technology, 7491 Trondheim, Norway*
[4] *Department of Chemistry, Purdue University, West Lafayette, IN 47907, USA*
[5] *Dipartimento di Chimica, Biologia e Biotecnologie, Università degli Studi di Perugia, Via Elce di Sotto, 8, 06123, Perugia, Italy*
[6] *Max Planck Institute for the Structure and Dynamics of Matter and Center Free-Electron Laser Science, Luruper Chaussee 149, 22761 Hamburg, Germany*
[7] *Scuola Normale Superiore, Piazza dei Cavalieri, 7, 56124 Pisa, Italy*¶

(Dated: September 1, 2023)

CONTENTS

I. *Ab Initio* Calculations S1

II. Perturbation Theory S2

III. Many-body Interactions S5

IV. Molecular Dynamics S6
  A. Training Potential Energy Functions for Simulating Fluids of H_2 S6
    1. Neural Network-based Pairwise Interactions S6
    2. Single Molecule Potential Energies S8
  B. Molecular Dynamics S8
    1. Classical Molecular Dynamics S8
    2. Path Integral Molecular Dynamics S8
  C. Radial Distribution Functions S9
  D. Angular Distribution Functions S9

V. Additional Results S9
  A. Comparison of Radial Distribution Functions S9
  B. Comparison of Classical MD and PIMD S10
  C. Comparison of QED-FCI-5 and QED-CCSD-12-SD1 S10
  D. λ Dependent Molecular Alignment S10

References S16

* jphilbin01@gmail.com
† chen4116@purdue.edu
‡ prineha@ucla.edu
§ henrik.koch@sns.it
¶ Denotes equal contribution

I. *AB INITIO* CALCULATIONS

The Hamiltonian used in the *ab initio* calculations is the single mode Pauli-Fierz Hamiltonian in the length gauge

$$H = H_e + \lambda\sqrt{\frac{\omega_c}{2}}((\boldsymbol{d} - \langle\boldsymbol{d}\rangle)\cdot\boldsymbol{\varepsilon})(b + b^\dagger) + \frac{\lambda^2}{2}((\boldsymbol{d} - \langle\boldsymbol{d}\rangle)\cdot\boldsymbol{\varepsilon})^2 + \omega_c b^\dagger b, \quad \text{(S1)}$$

where H_e is the electronic Hamiltonian, λ is the bilinear coupling, ω_c is the cavity frequency, $\boldsymbol{d}$ is the molecular dipole, $\boldsymbol{\varepsilon}$ is the cavity polarization vector, and b and $b^\dagger$ are the photon annihilation and creation operators, respectively.

All electronic structure calculations are run using an aug-cc-pVDZ basis set. The optical cavity is described by a single linearly polarized mode coupling parameter λ is set to 0.1 a.u. and the cavity energy $\hbar\omega_c$ is 13.6 eV, unless otherwise specified.

The large value for the coupling is partially justified by the single mode approximation. For cavity-induced changes in the ground state, each cavity mode will to second order in perturbation theory (see Eq. S12) enter the energy independently. For larger frequencies, the bilinear contribution from each mode cancels part of the dipole self-energy. For smaller frequencies compared to electronic excitation energies, we find that only contributions from the dipole self-energy are significant. Therefore, in the low-frequency regime, the coupling from N_{modes} modes is given by an effective coupling $\lambda_{\text{eff}}^2 \approx N_{\text{modes}}\lambda^2$.

As shown and discussed in Ref.[1], cavity quantum electrodynamics Hartree-Fock (QED-HF) and current QED density functional theory (QEDFT) implementations do not describe intermolecular forces properly, especially van der Waals interactions in

which they fail to predict an attractive interaction between van der Waals molecules. Therefore, we performed the *ab initio* simulations with QED coupled cluster (QED-CCSD-12-SD1) and QED full configuration interaction (QED-FCI).[2] QED-CCSD-12-SD1 is an extension of QED-CCSD-1, as described in Ref.[3], with two-photon excitations. The QED-CCSD-12-SD1 cluster operator is

$$T = T_1 + T_2 + S_1 b^\dagger + S_2 b^\dagger + \gamma_1 b^\dagger + \gamma_2 (b^\dagger)^2, \quad \text{(S2)}$$

where T_1 and T_2 are singles and doubles electron excitations, $S_1 b^\dagger$ and $S_2 b^\dagger$ are singles and doubles coupled electron-photon excitations, and $\gamma_1 b^\dagger$ and $\gamma_2 (b^\dagger)^2$ are singles and doubles photon excitations. The reference state is QED-HF as described in Ref.[3]. QED-FCI calculations are run with up to five photons (QED-FCI-5) to ensure that the energy with respect to photon number is converged.

We use QED-CCSD-12-SD1 instead of QED-CCSD-1 (equivalent to QED-CCSD-1-SD1) because the two-photon excitations are important for properly modeling the two-body interactions, as tested against QED-FCI-5 calculations. Without two-photon excitations, the two-body interactions have the wrong sign in the case of molecules separated by large distances (e.g. molecules separated by more than 1 nm). This is visualized in Fig. S1.

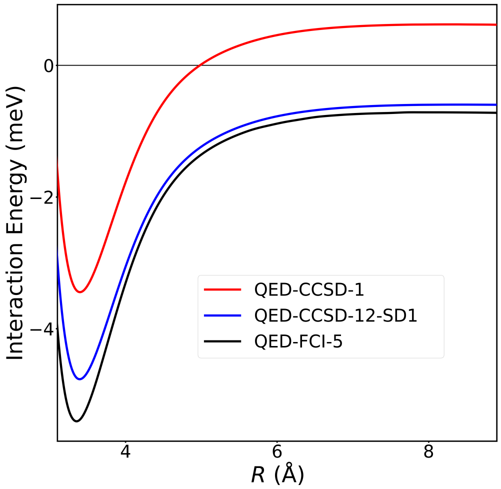

Fig. S1: Calculated intermolecular interaction energies for a C_{2v} configuration of $2H_2$ with the cavity polarization vector parallel to the center-to-center intermolecular distance vector. All calculations shown in this figure were performed with $\lambda = 0.1$ a.u

In all of our calculations, we use a linearly polarized optical cavity with a single photon frequency and single polarization vector. In most experiments as of today, the optical cavity is not limited to just one polarization, but rather it hosts two degenerate cavity modes with orthogonal polarizations (both cavity mode polarization vectors are perpendicular to the cavity wavevector). Since the molecular orientations aligns with the transversal polarization, we expect that a standard optical cavity, which has both polarizations, will interact with the system differently. In particular, we expect that for few molecules, the molecules will orient along the wavevector $\boldsymbol{k}$, perpendicular to both cavity polarization vectors. For many molecules, we expect that the molecules will align perpendicular to $\boldsymbol{k}$, in the plane defined from the two transversal polarization vectors.

II. PERTURBATION THEORY

As we demonstrate throughout this work, strong coupling to a single photon mode fundamentally changes the length scales and orientational dependence in which van der Waals molecules interact with one another. In this section, we explain these observations by performing perturbation theory in a similar spirit as Fritz London did in 1930[4–6] but with additional perturbative potentials associated with coupling to the cavity. This analysis shows cavity-mediated intermolecular interactions between van der Waals molecules that scale with R^{-3} and distance independent, R^0, interactions in addition to modifications to London dispersion forces that have an R^{-6} dependence.[7–10]

The total Hamiltonian is given by $H = H^0 + H^1$ with

$$H^0 = H_{e,A} + H_{e,B} + \omega_c b^\dagger b \quad \text{(S3)}$$

where $b^\dagger$ and b are photon creation and annihilation operators for the cavity mode of frequency ω_c and $H_{e,A}$ and $H_{e,B}$ refer to the electronic Hamiltonians of molecules A and B, respectively. The perturbative Hamiltonian (H^1) includes the dipolar coupling between molecules A and B, in the spirit of London's first derivation of van der Waals interactions, and the light-matter coupling to a single cavity mode

$$H^1 = -\frac{\boldsymbol{d}_A \cdot \boldsymbol{d}_B}{R^3} + \frac{3(\boldsymbol{d}_A \cdot \boldsymbol{R})(\boldsymbol{d}_B \cdot \boldsymbol{R})}{R^5} + \lambda\sqrt{\frac{\omega_c}{2}}(\boldsymbol{\varepsilon} \cdot \Delta\boldsymbol{d}_A + \boldsymbol{\varepsilon} \cdot \Delta\boldsymbol{d}_B)(b + b^\dagger) + \frac{\lambda^2}{2}(\boldsymbol{\varepsilon} \cdot \Delta\boldsymbol{d}_A + \boldsymbol{\varepsilon} \cdot \Delta\boldsymbol{d}_B)^2 \quad \text{(S4)}$$

where $\Delta\boldsymbol{d}_A = \boldsymbol{d}_A - \langle\boldsymbol{d}_A\rangle$ and $\Delta\boldsymbol{d}_B = \boldsymbol{d}_B - \langle\boldsymbol{d}_B\rangle$ are the fluctuations of molecule A and molecule B's dipoles, respectively and $\boldsymbol{d}_A$ and $\boldsymbol{d}_B$ are the dipole operators for molecule A and molecule B, respectively. Recall that in this work we are working with van der Waals molecules such that both molecules do not have permanent dipoles (i.e. $\langle\boldsymbol{d}_A\rangle = \langle\boldsymbol{d}_B\rangle = 0$).

The first-order correction to the energy is given by

$$E^1 = \langle g|H^1|g\rangle \quad \text{(S5)}$$

where $|g\rangle$ denotes the ground state of the total system, $|g\rangle = |g_A\rangle\,|g_B\rangle\,|g_c\rangle$ where molecule A, molecule B, and the cavity are in their ground states. In this illustrative perturbation theory, we are interested in the asymptotic behavior for when molecule A and molecule B are far away from one another; thus, the antisymmetry of the total electronic wavefunctions is ignored. Substituting in Eq. S4 into Eq. S5, we obtain

$$\begin{aligned} E^1 &= \frac{\lambda^2}{2}\left(\langle g_A|(\boldsymbol{d_A} \cdot \boldsymbol{\varepsilon})^2|g_A\rangle + \langle g_B|(\boldsymbol{d_B} \cdot \boldsymbol{\varepsilon})^2|g_B\rangle\right) \\ &= \frac{\lambda^2}{2}(E_A^1 + E_B^1) \end{aligned} \quad \text{(S6)}$$

where $E_A^1 = \langle g_A|(\boldsymbol{d_A} \cdot \boldsymbol{\varepsilon})^2|g_A\rangle$ and $E_B^1 = \langle g_B|(\boldsymbol{d_B} \cdot \boldsymbol{\varepsilon})^2|g_B\rangle$ are the dipole self-energies of molecule A and molecule B, respectively. In Eq. S6 we have used the facts that there are no photons in the ground state of the cavity ($\langle g_c|b^\dagger b|g_c\rangle = 0$) and that for van der Waals molecules, by definition, there is no permanent dipole ($\langle g_A|\boldsymbol{d}_A|g_A\rangle = \langle\boldsymbol{d}_A\rangle = 0$ and $\langle g_B|\boldsymbol{d}_B|g_B\rangle = \langle\boldsymbol{d}_B\rangle = 0$). The fact that molecules A and B do not have permanent dipoles allows us to express E_A^1 and E_B^1 with a different formula, i.e.

$$\begin{aligned} E_A^1 &= \langle g_A|(\boldsymbol{d_A} \cdot \boldsymbol{\varepsilon})^2|g_A\rangle \quad \text{(S7)} \\ &= \langle g_A|(\boldsymbol{d_A} \cdot \boldsymbol{\varepsilon})\,\hat{I}\,(\boldsymbol{d_A} \cdot \boldsymbol{\varepsilon})|g_A\rangle \\ &= \sum_{e_A} |\,\langle e_A|(\boldsymbol{d_A} \cdot \boldsymbol{\varepsilon})|g_A\rangle\,|^2 \ , \end{aligned}$$

where $|e_A\rangle$ is an excited state of molecule A. An important observation here is that both E_A^1 and E_B^1 are single molecule terms and are always positive; we will return to these facts after deriving the second-order energy correction.

The second-order correction to the energy is given by

$$E^2 = -\sum_e \frac{\left|\,\langle e|H^1|g\rangle\right|^2}{E_e - E_g} \quad \text{(S8)}$$

where $|g\rangle$ is the ground state of the bi-molecule system with energy E_g and $|e\rangle$ indicates an excited state of the bi-molecule system with energy E_e. Substituting Eq. S4 into Eq. S8 along with some simplifications we obtain the second-order correction to the energy to be

$$E^2 = -\sum_{e_A e_B} \frac{|\langle e_A e_B | V_{AB} | g_A g_B\rangle|^2}{E_{e_A} - E_{g_A} + E_{e_B} - E_{g_B}} - \lambda^2 \sum_{e_A e_B} \frac{\langle e_A e_B | V_{AB} | g_A g_B\rangle\ \langle e_A | \boldsymbol{d_A} \cdot \boldsymbol{\varepsilon} | g_A\rangle\ \langle e_B | \boldsymbol{d_B} \cdot \boldsymbol{\varepsilon} | g_B\rangle}{E_{e_A} - E_{g_A} + E_{e_B} - E_{g_B}}$$
$$- \frac{\lambda^2 \omega_c}{2} \left[\sum_{e_A} \frac{|\langle e_A | \boldsymbol{d_A} \cdot \boldsymbol{\varepsilon} | g_A\rangle|^2}{\omega_c + E_{e_A} - E_{g_A}} + \sum_{e_B} \frac{|\langle e_B | \boldsymbol{d_B} \cdot \boldsymbol{\varepsilon} | g_B\rangle|^2}{\omega_c + E_{e_B} - E_{g_B}} \right]$$
$$- \frac{\lambda^4}{4} \left[\sum_{e_A} \frac{\left|\langle e_A | (\boldsymbol{d_A} \cdot \boldsymbol{\varepsilon})^2 | g_A\rangle\right|^2}{E_{e_A} - E_{g_A}} + \sum_{e_B} \frac{\left|\langle e_B | (\boldsymbol{d_B} \cdot \boldsymbol{\varepsilon})^2 | g_B\rangle\right|^2}{E_{e_B} - E_{g_B}} + 4 \sum_{e_A e_B} \frac{|\langle e_A | (\boldsymbol{d_A} \cdot \boldsymbol{\varepsilon}) | g_A\rangle|^2\, |\langle e_B | (\boldsymbol{d_B} \cdot \boldsymbol{\varepsilon}) | g_B\rangle|^2}{E_{e_A} - E_{g_A} + E_{e_B} - E_{g_B}} \right]$$
$$= E^2_{AB,d^0} + \lambda^2 E^2_{AB,d^1} + \frac{\lambda^2}{2}(E^2_{A,d^1} + E^2_{B,d^1}) + \frac{\lambda^4}{4}(E^2_{A,d^2} + E^2_{B,d^2} + E^2_{AB,d^2}) \tag{S9}$$

where we defined

$$V_{AB} = -\frac{\boldsymbol{d}_A \cdot \boldsymbol{d}_B}{R^3} + \frac{3(\boldsymbol{d}_A \cdot \boldsymbol{R})(\boldsymbol{d}_B \cdot \boldsymbol{R})}{R^5} \quad . \tag{S10}$$

E^2_{AB,d^0}, E^2_{AB,d^1}, E^2_{A,d^1}, E^2_{B,d^1}), E^2_{A,d^2}, E^2_{B,d^2}, and E^2_{AB,d^2} are defined as

$$E^2_{AB,d^0} = -\sum_{e_A e_B} \frac{|\langle e_A e_B | V_{AB} | g_A g_B\rangle|^2}{E_{e_A} - E_{g_A} + E_{e_B} - E_{g_B}} \tag{S11a}$$

$$E^2_{AB,d^1} = -\sum_{e_A e_B} \frac{\langle e_A e_B | V_{AB} | g_A g_B\rangle\ \langle e_A | \boldsymbol{d_A} \cdot \boldsymbol{\varepsilon} | g_A\rangle\ \langle e_B | \boldsymbol{d_B} \cdot \boldsymbol{\varepsilon} | g_B\rangle}{E_{e_A} - E_{g_A} + E_{e_B} - E_{g_B}} \tag{S11b}$$

$$E^2_{A,d^1} = -\omega_c \sum_{e_A} \frac{|\langle e_A | \boldsymbol{d_A} \cdot \boldsymbol{\varepsilon} | g_A\rangle|^2}{\omega_c + E_{e_A} - E_{g_A}} \tag{S11c}$$

$$E^2_{B,d^1} = -\omega_c \sum_{e_B} \frac{|\langle e_B | \boldsymbol{d_B} \cdot \boldsymbol{\varepsilon} | g_B\rangle|^2}{\omega_c + E_{e_B} - E_{g_B}} \tag{S11d}$$

$$E^2_{A,d^2} = -\sum_{e_A} \frac{\left|\langle e_A | (\boldsymbol{d_A} \cdot \boldsymbol{\varepsilon})^2 | g_A\rangle\right|^2}{E_{e_A} - E_{g_A}} \tag{S11e}$$

$$E^2_{B,d^2} = -\sum_{e_B} \frac{\left|\langle e_B | (\boldsymbol{d_B} \cdot \boldsymbol{\varepsilon})^2 | g_B\rangle\right|^2}{E_{e_B} - E_{g_B}} \tag{S11f}$$

$$E^2_{AB,d^2} = -4 \sum_{e_A e_B} \frac{|\langle e_A | (\boldsymbol{d_A} \cdot \boldsymbol{\varepsilon}) | g_A\rangle|^2\, |\langle e_B | (\boldsymbol{d_B} \cdot \boldsymbol{\varepsilon}) | g_B\rangle|^2}{E_{e_A} - E_{g_A} + E_{e_B} - E_{g_B}} \quad , \tag{S11g}$$

where $|g_A\rangle$ ($|g_B\rangle$) is the ground state of molecule A (B) with energy E_{g_A} (E_{g_B}), $|e_A\rangle$ ($|e_B\rangle$) indicates an excited state of molecule A (B) with energy E_{e_A} (E_{e_B}), and $\langle e_A|\boldsymbol{d}_A|g_A\rangle$ ($\langle e_B|\boldsymbol{d}_B|g_B\rangle$) is the transition dipole moment of molecule A (B) associated with the excited state. Eq. S9 is an important result in this work, and the physical interpretation, origin, and implications of each term are worth exploring in detail. E^2_{AB,d^0} in Eq. S9 is the typical attractive London dispersion interaction with its prototypical R^{-6} dependence (as each V_{AB} scales with R^{-3}). The remaining terms all arise from in-

teractions through the cavity mode. E^2_{AB,d^1} contains a single V_{AB} matrix element giving an R^{-3} of this term. Interestingly, this term also contains dot products of transition dipole moments ($\langle e_A|\boldsymbol{d}_A|g_A\rangle$) with the cavity polarization vector ($\boldsymbol{\varepsilon}$). This R^{-3} term is central to this work as it says that van der Waals molecules inside a cavity have this interesting interaction length scale that also has unique, coupled molecule-molecule and molecular-cavity angle dependencies. E^2_{A,d^1} and $E^2_{B,d^1}$are very similar to E^1_A and E^1_B except that E^2_{A,d^1} and E^2_{B,d^1} arise from the bilinear coupling term and have the opposite sign as E^1_A and E^1_B. Specifically, to second-order in the coupling λ, the one-body energy (e.g. molecule A) is given by

$$\begin{aligned} E_A^{\text{cavity}} &= E_A^{\text{no cavity}} + \frac{\lambda^2}{2}(E^1_A + E^2_{A,d^1}) \qquad \text{(S12)} \\ &= E_A^{\text{no cavity}} + \frac{\lambda^2}{2}\sum_{e_A} |\langle e_A|\boldsymbol{d}_A\cdot\boldsymbol{\varepsilon}|g_A\rangle|^2 \\ &- \frac{\lambda^2\omega_c}{2}\sum_{e_A}\frac{|\langle e_A|\boldsymbol{d}_A\cdot\boldsymbol{\varepsilon}|g_A\rangle|^2}{w_c + E_{e_A} - E_{g_A}} \ . \end{aligned}$$

A similar energy term can be derived for molecule B as well. We want to emphasize that E^1_A arises from the dipole self-energy term in first-order perturbation theory (Eq. S6) and E^2_{A,d^1} arises from the bilinear coupling term in second-order perturbation theory (Eq. S9). Interestingly, E^1_A and E^2_{A,d^1} only exactly cancel if the cavity frequency is much larger than the electronic transition energies ($\omega_c \gg E_{e_A} - E_{g_A}$). Thus, for H_2 molecules with a cavity in the electronic regime ($\omega_c = 13.6$ eV here) the total energy of a single molecule ends up increasing with λ^2 (main text Fig. 3A). For H_2 molecules, the one-body energy reaches a minimum when the molecular bond is perpendicular to the cavity polarization vector ($\theta_{A\varepsilon} = \frac{\pi}{2}$). Intuitively, this occurs because H_2 is most polarizable along its bond axis which leads to $\sum_{e_A} |\langle e_A|\boldsymbol{d}_A\cdot\boldsymbol{\varepsilon}|g_A\rangle|^2/(E_{e_A} - E_{g_A}) = \boldsymbol{\varepsilon}^T\boldsymbol{\alpha}\boldsymbol{\varepsilon}$ being largest when $\theta_{A\varepsilon} = 0, \pi$.

E^2_{A,d^2}, E^2_{B,d^2}, and E^2_{AB,d^2} arise from two factors of the dipole self-energy part of Eq. S4 and, thus, scale with λ^4. While E^2_{A,d^2} and E^2_{B,d^2} are corrections to the one-body energies, E^2_{AB,d^2} impacts the two-body energies (i.e. intermolecular interaction energy). Furthermore, this term has no R dependence, and, thus, E^2_{AB,d^2} is the first term that we have discussed that gives rise to the collective orientational order reported in the main text. The magnitude of this term is greatest when both molecules have their bonds oriented along the cavity polarization vector ($\boldsymbol{\varepsilon}$), because $\boldsymbol{\varepsilon}^T\boldsymbol{\alpha}_A\boldsymbol{\varepsilon}$ and $\boldsymbol{\varepsilon}^T\boldsymbol{\alpha}_B\boldsymbol{\varepsilon}$ are both largest in the case which both of their bonds are oriented parallel to $\boldsymbol{\varepsilon}$. And because of the negative sign in front of this infinite range interaction term, it contributes to lowering the energy of molecular configurations in which the molecular bonds of the hydrogen molecules are oriented parallel to the cavity polarization vector, as shown in Fig. 3C of the main text.

III. MANY-BODY INTERACTIONS

The many-body expansion,

$$E = \sum_A E_A + \sum_{AB} E_{AB} + \sum_{ABC} E_{ABC} + \dots \qquad \text{(S13)}$$

is a routinely used expansion for modeling and gaining insight into intermolecular forces.[11] For van der Waals type intermolecular forces, the higher-order interactions such as E_{ABC} quickly become negligible with distance and they can be assumed to be much smaller than the lower-order terms at large distances. QED electronic structure calculations allow us to test if the three-body and higher-order terms can be ignored for the strong light-matter coupling cavity QED Hamiltonian with similar parameters used in the calculations of the main text. In Table S1 and Fig. S2, we show the intermolecular interactions for molecules separated far apart, 25 Å. As expected, QED-HF does not capture the dynamic correlation and cannot describe the intermolecular forces arising from neither the cavity nor the van der Waals forces. QED-CCSD-1 captures the dynamic correlation, but the sign of the two-body interaction is not consistent with QED-FCI. Adding just one more term to the cluster operator of QED-CCSD-1, the two-photon $(b^\dagger)^2$ term in QED-CCSD-12-SD1, yields a sufficient description of the two-body interactions. For QED-CCSD-12-SD1, we find that the higher-order terms quickly approach zero even for the very strong coupling $\lambda = 0.1$ a.u. From perturbation theory, we find that the N-body interactions are sensitive to the light-matter coupling strength and scale as λ^{2N} (see Fig. S2).

A few additional key points about the many-body expansion of van der Waals interactions in the context of the nonrelativistic cavity QED Hamiltonian given in Eq. S1 are worth mentioning here. Because the three-body interactions have opposite sign to the two-body interactions (Table S1), we expect that the collective orientational order induced by the infinite range cavity-induced interactions would be reduced by including the three-body terms in the molecular dynamics simulations. While the three-body terms are insignificant on a per interaction basis, the lack of distance (R) dependence in the cavity-induced interactions, see Eq. S9, results in all molecules in the simulation interacting with all other

molecules independent of how far away they are from each other. In a simulation with n molecules, there are $n(n-1)/2 \sim n^2$ two-body interactions, $n(n-1)(n-2)/6 \sim n^3$ three-body interactions, and similarly for higher-order terms (Table S2). Therefore, there must exist a number of molecules where the total three-body energy is larger than the total two-body energy. This makes it very challenging to extrapolate our results to truly macroscopic systems. Extending these microscopic equations and calculations to truly macroscopic systems remains an open question.

Method	1-body	2-body	3-body	4-body
QED-HF	204.9	0.0000	0.0000	0.0000
QED-CCSD-1	107.5	0.3238	-0.0571	0.0042
QED-CCSD-12-SD1	107.1	-0.5600	0.0104	-0.0004
QED-FCI-5	106.7	-0.6601	...	...

Table S1: Cavity-induced N-body effects for different QED electronic structure methodologies with $\lambda = 0.1$ a.u. The cavity energy is $\hbar\omega_c = 13.6$ eV and polarization perpendicular to all molecules. The molecules are placed on the edges of a line (E_{AB}), equilateral triangle (E_{ABC}) and square (E_{ABCD}), all with side lengths of 25 Å. All numbers in the table are meV. QED-FCI-5 is too computationally expensive for more than two H_2 molecules in the aug-cc-pVDZ basis set.

	1-body	2-body	3-body	4-body
Scaling with coupling	λ^2	λ^4	λ^6	λ^8
Number of terms	$\binom{n}{1}$	$\binom{n}{2}$	$\binom{n}{3}$	$\binom{n}{4}$

Table S2: The number of interactions and scaling of the cavity-induced interaction energy in the Nth body of the N-body expansion for a system with n molecules.

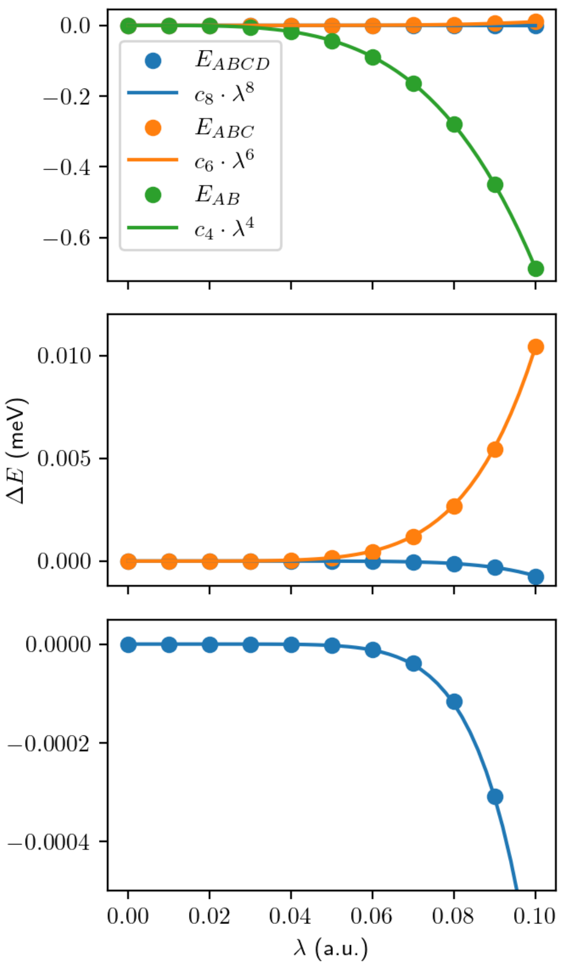

Fig. S2: N-body effects for different coupling strengths λ. All calculations are performed on N H_2 molecules with QED-CCSD-12-SD1. The cavity energy is $\hbar\omega_c = 13.6$ eV and polarization perpendicular to all molecules. The molecules are placed on the edges of a line (E_{AB}), equilateral triangle (E_{ABC}) and square (E_{ABCD}), all with side lengths of 25 Å.

IV. MOLECULAR DYNAMICS

A. Training Potential Energy Functions for Simulating Fluids of H_2

1. *Neural Network-based Pairwise Interactions*

We developed neural network-based potential energy functions (NNPs) for the pairwise interaction of a pair of hydrogen molecules using *ab initio* energy data with CCSD, FCI, QED-CCSD-12-SD1, and QED-FCI levels of theory. The potential energy functions have the forms,

$$E_{\mathrm{AB}}^{\text{no cavity}} = c_{\exp} \exp(-aR) - \frac{c_6\{\theta\}}{R^6} \tag{S14}$$

$$E_{\mathrm{AB}}^{\text{cavity}} = E_{\mathrm{2b}}^{\text{no cavity}} - \frac{c_3\{\theta\}}{R^3} + \frac{c_0\{\theta\}}{R^0} \tag{S15}$$

where $c_{\exp}, a, c_6, c_3, c_0$ are represented by neural networks (NNs). Each NN takes symmetry preserved features of a pair of molecules as input. Symmetry preserved features that have been selected as the input for the machine learning (ML) model to get the pairwise interaction energy are shown pictorially in Fig. S3 and are listed in Table S3. In the case without the cavity field, the interaction energies are obtained using the input features

$\theta_{\mathbf{R}A}, \theta_{\mathbf{R}B}, \theta_{AB}, \|\mathbf{R}\|$. For the cavity case, additional terms that depend on the cavity polarization vector are added. In particular, $\theta_{A\varepsilon}, \theta_{B\varepsilon}$, and $\theta_{\mathbf{R}\varepsilon}$ are added and $\|\mathbf{R}\|$ is replaced by R_{cap} and $R_{\text{cap}} = \mathrm{C}\tanh(\|\mathbf{R}\|/\mathrm{C})$, where C is a cutoff distance. In order to account for molecular and exchange symmetries, $\cos 2\theta$ and $\sin 2\theta$ are used for any $\theta \in \Theta \equiv \{\theta_{\mathbf{R}A}, \theta_{\mathbf{R}B}, \theta_{\text{AB}}, \theta_{A\varepsilon}, \theta_{B\varepsilon}, \theta_{\mathbf{R}\varepsilon}\}$. For each of c_{exp}, a, c_6, c_3, we are using $\mathrm{F}(\Theta, R_{\text{cap}})+\mathrm{F}(\tilde{\Theta}, \tilde{R}_{\text{cap}})$ where $\tilde{\Theta}$ and $\tilde{R}_{\text{cap}}$ are calculated by switching the index of the two molecules. For c_0, only Type 1 features as tabulated in Table S3 were used.

The neural network model has four fully-connected layers including a linear output layer. The other three linear layers have CELU activation functions.[12] The number of neurons per layer is 64 in our model. To train the model, we used energy data points of pair configurations that are generated using a classical MD simulation of liquid H_2. 10^5 pair configurations generated by MD simulation were used to compute energies with CCSD level of theory for training model when no cavity is present. While the pair configurations generated by MD simulation were good enough to train a model without a cavity, long range pair configurations are extremely important to train the model with a cavity. Similarly, short range pair configurations are very crucial to accurately reproduce the corrected short range repulsion energies in the potential energy functions in the presence of a cavity. While MD of liquid H_2 produces good random configurations with various possible orientations, the probability of finding short range pair configurations is low in an MD simulation. In order to include sufficient number of configurations at short range, we randomly select 10% of the total configurations obtained from MD simulation of liquid H_2 molecules and scale the intermolecular distance to be within $2-5$ Å. A similar strategy was followed to generate very long range configurations between $18-90$ Å for 10% of the total configurations. A total of 121,000 data points, including both the additional short range and long range configurations, were used to the train the NN model to the QED-CCSD-12-SD1 calculated energies in the cavity case. For training using the QED-FCI calculated data, we use a smaller data set of 30,000 calculated energies. In order to train the model on this smaller data set, we initialize each NN with the parameters obtained from our QED-CCSD-12-SD1 fits, which was trained using a larger data set of 121,000 calculated energies. We use the Adam optimizer[13] with $\beta_1 = 0.90$ and $\beta_2 = 0.99$. And we utilize a constant learning rate of 10^{-5} and a batch size of 32. 90% of the total data points were used in the training data set and the remaining 10% were used as a test data set. All training and testing protocols were implemented with PyTorch.[14]

Type of feature	Features
Type 1	$\cos 2\theta_{A\varepsilon}$, $\sin 2\theta_{A\varepsilon}$,$\cos 2\theta_{B\varepsilon}$, $\sin 2\theta_{B\varepsilon}$
Type 2	$\cos 2\theta_{\mathbf{R}\varepsilon}$, $\sin 2\theta_{\mathbf{R}\varepsilon}$, $\cos 2\theta_{\mathbf{R}A}$, $\sin 2\theta_{\mathbf{R}A}$ $\cos 2\theta_{\mathbf{R}B}$, $\sin 2\theta_{\mathbf{R}B}$, $\cos 2\theta_{AB}$, $\sin 2\theta_{AB}$
Type 3	$\mathrm{C}\tanh(\|\mathbf{R}\|/\mathrm{C})$

Table S3: Input features involved in the energy contributions.

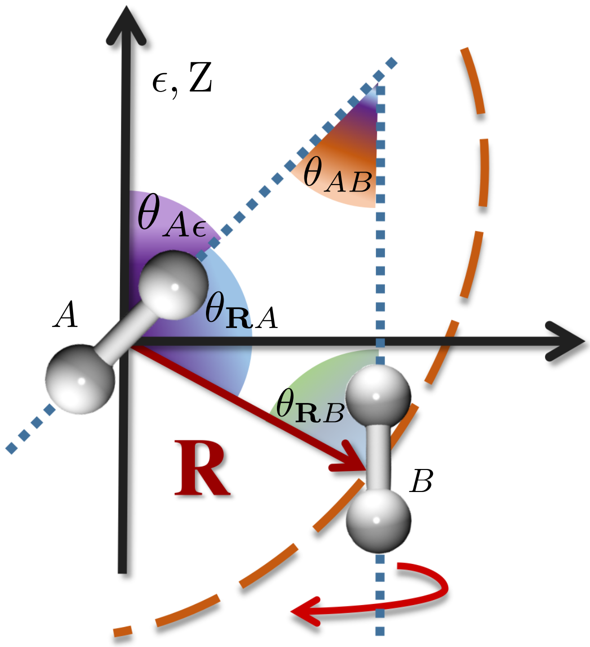

Fig. S3: Symmetry preserved features that are considered while generating the pair interaction potential using a neural network based machine learning model are shown here. Various angles between a pair of molecule which are considered as input features are shown. **R** is the distance vector of the center of mass (COM) of molecule A and molecule B. ε represents the cavity polarization. Orientation of the molecules are completely specified by various angles $\{\theta\}$.

The energies of the *ab initio* (CCSD) calculations and the ML predicted energies of the pairs of molecules without a cavity field are shown in the Fig. S9A. A linearity plot shows the accuracy of the predicted energy using our ML model. Apart from the linearity plot, we scanned potential energy curves for a few selected orientations of pairs of molecules. These results show that the ML predicted potential energy curves for pairs of hydrogen molecules are in good agreement with the potential energy curves obtained from *ab initio* calculations. These plots are shown in Fig. S9B. A linearity plot comparing the

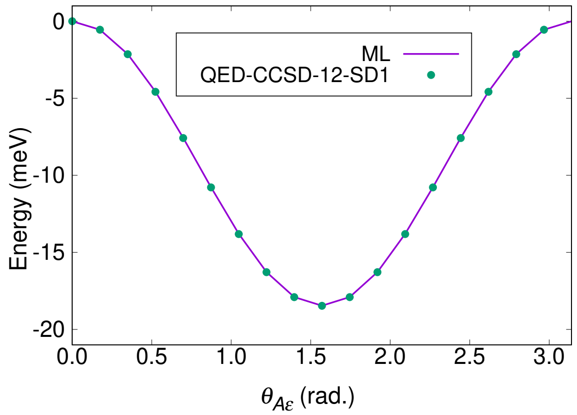

Fig. S4: Energy of a single H_2 molecule inside a cavity with respect to cavity polarization vector, ε using it ab initio QED-CCSD-12-SD1 and ML. Single molecular energy at $\varepsilon = 0.0$ was set to zero while plotting energies of both QED-CCSD-12-SD1 and ML.

ab initio (QED-CCSD-12-SD1) calculations and the ML predicted energies with the cavity field turned on are shown in Fig. S10A. Potential energy curves (Fig. S10B) were scanned for D_{2h} configuration of a pair of molecules along three different cavity polarization directions with respect to the molecular bond axis. These plots shows that our ML model accurately reproduces the *ab initio* potential energy curves.

2. *Single Molecule Potential Energies*

Single molecule potential energies involve intra-molecular chemical bonds and the cavity-modified single molecule contributions. Intra-molecular chemical bonds were modeled within the harmonic approximation. We like to emphasize that the intra-molecular interaction energy does not play a significant role in determining the properties that we focused on in this study.

Single molecule energies in the presence of a cavity field is important. Training of the cavity-modified single molecule energies has been done with a linear regression method. The following form of energy function is trained for the single molecule energies,

$$E_{\mathrm{A}} = \sum_{n=1}^{3} C_n \sin 2n\theta + \sum_{n=0}^{2} D_n \cos 2n\theta \tag{S16}$$

where θ is the angle between the molecular bond axis and the cavity polarization vector. C_n and D_n are the trainable parameters. Fig. S4 shows the accuracy of fitting single molecular energies with respect to the *ab initio*, QED-CCSD-12-SD1 calculations.

B. Molecular Dynamics

Molecular dynamics (MD) simulations were used to compute the statistical properties of fluids of H_2 molecules at 70 K by employing the potential energy functions, generated by our machine learning models. For computing the statistical behaviour of the system both classical MD and path integral MD (PIMD) were used.

1. *Classical Molecular Dynamics*

NVT ensemble MD simulations were carried out using Langevin dynamics with a time step of 1.0 femtosecond (fs) and the friction coefficient for the Langevin dynamics was chosen 0.0005 a.u (20.7 ps^{-1}). Random initial atomic velocities and random initial positions were provided to run MD. In order to use ML potentials generated with PyTorch, we also implement the MD engine with PyTorch. The integrator used here is described in Ref.[15]. Forces were computed using the PyTorch autograd module and the PyTorch MD simulations were performed using GPUs.

Since we are simulating a fluid system, the system was confined within a spherical volume, similar to a cluster of molecules. In practice, a stiff harmonic potential was used to confine the center of the mass of each molecule within a spherical volume with radius R_{c} (see Fig. S5). Adopting such a boundary condition was necessary in order to account for non-decaying nature of the pair interaction potential inside of an optical cavity. In order to simulate various different system sizes, R_{c} is scaled appropriately to preserve the overall molecular density.

2. *Path Integral Molecular Dynamics*

In the previous section, we discussed the MD simulations in which the nuclei were considered as classical particles. However, for light nuclei such as hydrogen atoms, this assumption could lead to serious problems in predicting the statistical properties because of strong quantum nuclei effects, especially at low temperatures. In order to account for quantum nuclei effects in our MD simulations, we performed path integral molecular dynamics (PIMD) simulations.

Usually PIMD simulations require a large number of beads to converge thermodynamics properties at

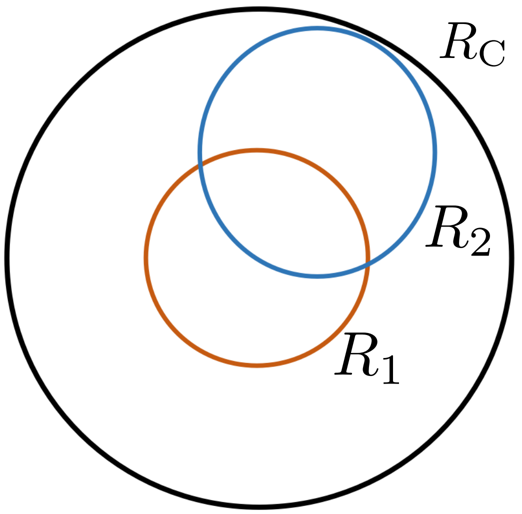

Fig. S5: Schematic diagram of the radius cutoff that are used in computing radial distribution functions. R_c is the distance at which a high energy potential barrier has been applied. R_1 is the radius of core region where surface effects due to the spherical boundary are minimal and molecules found within the radius of R_2 are used to compute the histogram of pairwise distance for the calculations of the radial distribution functions.

low temperatures. Herein, we used the generalized Langevin equation (GLE) in PIMD, which can significantly reduce the number of beads.[16–18] In the GLE formulation,[19] each bead of the simulated system is coupled to several extended degrees of freedom with an appropriate drift matrix and a diffusion matrix to approximate a friction kernel function. We used 8 extra degrees of freedom in GLE and the drift matrix and diffusion matrix used in GLE were generated by an online tool called GLE4MD (http://gle4md.org/) with the maximum physical frequency set to $\omega_{\max} = 9608$ cm^{-1}. With the GLE formulation, we observed that using 32 beads are able to converge the simulations whereas more than 128 beads are needed to converge the results without the GLE formulation. We have developed an interface to i-PI[20] to run the PIMD simulations using our ML potentials.

C. Radial Distribution Functions

The radial distribution functions (g(r)) of fluid of H_2 molecules are computed from the PIMD trajectories of $1,000$ molecules. As the system we simulated has a spherical volume without any periodic boundary, computing a bulk-like g(r) (i.e. a g(r) that converges to 1 in the long distance limit) is not straightforward. In order to compute g(r) from such a spherical system, the following steps are taken. First, a bulk-like core region is chosen within a certain cutoff distance R_1.

$$\bar{h}(|\mathbf{r}|) = \frac{1}{N_1} \sum_{i, R_i < R_1} h(|\mathbf{r} - \mathbf{r}_i|) \quad \text{(S17)}$$

For the ith molecule located at $\mathbf{r}_i$ with $R_i = |\mathbf{r}_i| < R_1$, $h(|\mathbf{r} - \mathbf{r}_i|)$ is the histogram of all distance between any other molecules and the ith molecule with $(|\mathbf{r} - \mathbf{r}_i|) < R_2$, $R_1 + R_2 < R_c$ and N_1 is the number of molecules inside R_1. Second, the average over each frame of MD or PIMD as well as the average over number of beads was computed in the calculations of the radial distribution functions. Lastly, the averaged $\bar{h}(|\mathbf{r}|)$ was normalized by the average density and $4\pi r^2$. In this study, $R_1 = 6.0$ Å and $R_2 = 12$ Å was used.

D. Angular Distribution Functions

We also computed angular distribution functions for the angle between the molecular bond axis of molecule A and the molecular bond axis of molecule B (θ_{AB}) and angular distribution functions for the angle between the molecular bond axis of molecule A and the cavity polarization vector ($\theta_{\mathrm{A}\varepsilon}$). The probability distributions of θ_{AB} and $\theta_{\mathrm{A}\varepsilon}$ are proportional to $\sin(\theta_{\mathrm{AB}})$ and $\sin(\theta_{\mathrm{A}\varepsilon})$, respectively, if molecules A and B can rotate freely without any interactions. In order to emphasize the energy contribution, we computed the potentials of mean force by scaling the probability distributions of θ_{AB} and $\theta_{\mathrm{A}\varepsilon}$ with their corresponding sine functions. In the case of PIMD, the average over each frame and the average over the number of beads are considered when computing the histograms.

V. ADDITIONAL RESULTS

A. Comparison of Radial Distribution Functions

We compute the radial distribution function at three different situations when (1) cavity polarization is not active, (2) cavity-modified one-body term is active but cavity modified two-body term is not active, and (3) both cavity modified one-body and two-body terms are active. We have observed differentiable changes in radial distribution function for three different situations. This indicates the difference in equilibrium structure when cavity polarization is on. The results are shown in Fig. S12.

B. Comparison of Classical MD and PIMD

In this section we compare the results of our classical MD and the PIMD simulations with $\lambda = 0.1$ a.u. Based on Fig. S6, it is evident that classical MD and PIMD qualitatively follow the same trend when angular distribution function of $\theta_{\mathrm{A}\varepsilon}$ and θ_{AB} are compared. In particular, one observes a strong orientational alignment of the molecules along direction of the cavity polarization vector occurring inside of an optical cavity. Inclusion of nuclear quantum effects does not change the overall conclusion. However, the extent of alignment of the molecules inside the cavity in our PIMD simulations is considerably reduced compared to our classical MD simulations.

C. Comparison of QED-FCI-5 and QED-CCSD-12-SD1

Here we compare our results of classical MD simulations using the ML potentials obtained from QED-FCI-5 and QED-CCSD-12-SD1 calculations. As summarized in Fig. S7, we see that classical MD with ML potentials that are obtained from the two different levels of *ab initio* calculations qualitatively match each other. However, the intensities in the angular distribution functions of $\theta_{\mathrm{A}\varepsilon}$ and θ_{AB} for the two cases are different. These differences are due to the quantitative differences in predicting the interaction energies using these two methods (see Fig. S1).

D. λ Dependent Molecular Alignment

Two different λ values were considered in our study. In the main text, we focused our discussion on the results with $\lambda = 0.1$ a.u. In this section, we study the properties of a system with $\lambda = 0.02$ a.u. and compare these results with the results obtained using $\lambda = 0.1$ a.u.

In order to train a model with $\lambda = 0.02$ a.u. important NN parameters for c_0 and c_3 were transferred and scaled from our training model with $\lambda = 0.1$ a.u. together with the perturbation theory analysis. The accuracy of the model has been tested by plotting the energies obtained from the NNPs against the *ab initio* energies. A linearity plot is obtained as shown in Fig. S11A. Additionally, scanned potential energy curves of several selected pair configurations are in good agreement with *ab initio* potential energy curves. Some of these plots are shown in Fig. S11B. The accuracy of our ML model is further justified with in Fig. S11C, where we show that our ML model correctly predicts the long range interaction energy with different directions of the cavity polarization vector.

A significant difference in the angular distribution functions of $\theta_{\mathrm{A}\varepsilon}$ is observed when the results of two different λ values are compared for $1,000$ H_2 molecules. The distribution function of $\theta_{\mathrm{A}\varepsilon}$ for $1,000$ H_2 molecules with $\lambda = 0.02$ a.u. (Fig. S8A) shows molecular alignment perpendicular to the cavity polarization ($\theta_{\mathrm{A}\varepsilon} = \frac{\pi}{2}$). On the other hand, we observe in Fig. S6A that the angular distribution function of $\theta_{\mathrm{A}\varepsilon}$ is maximized in the direction of cavity polarization vector ($\theta_{\mathrm{A}\varepsilon} = 0, \pi$) when $\lambda = 0.1$ a.u. This can be explained from our perturbation theory analysis where we showed that the cavity-modifications to the single molecule energies scale with λ^2 and the extremely long range pairwise interaction scales with λ^4. Thus, the importance of the pairwise interaction decreases much faster than the single molecule energy contribution as λ decreases. In this particular example of $1,000$ H_2 molecules with $\lambda = 0.02$ a.u., the single molecule energy dominates whereas, with $\lambda = 0.1$ a.u., the pairwise interaction energy dominates. θ_{AB} qualitatively follow the same trend as we observed for $1,000$ H_2 molecules with $\lambda = 0.1$ a.u.; however, the intensity of the peak is reduced which suggests a weaker synchronization of molecular orientations. This is shown in the inset of Fig. S8A.

From the above discussion, we understand that the energy contributions from a single molecule can be altered by (1) changing the number of molecules with a fixed λ, and (2) changing the value of λ for a fix number of molecules. We ran simulations considering these two possibilities. For the first possibility, we reduced the number of molecules from $1,000$ to 108 while keeping λ equal to 0.1 a.u., and we compute the angular distribution function for $\theta_{\mathrm{A}\varepsilon}$. We find that in the 108 molecule simulation the preferential alignment of the molecules is perpendicular to the cavity polarization vector, which is opposite to the alignment of $1,000$ molecules with $\lambda = 0.1$ a.u. (aligned parallel to the cavity polarization vector). These results are shown in Fig. S6A and Fig. S8B. For the second possibility, we simulate $1,000$ molecules with a reduced value of $\lambda = 0.02$ a.u. The angular distribution function of $\theta_{\mathrm{A}\varepsilon}$ in this simulation is qualitatively similar to the results obtained in the first possibility with the molecular alignment perpendicular to the cavity polarization vector (see Fig. S6A and Fig. S8B). All of our numerical simulation results reported in this section further confirm the conceptual validity of our perturbation theory analysis.

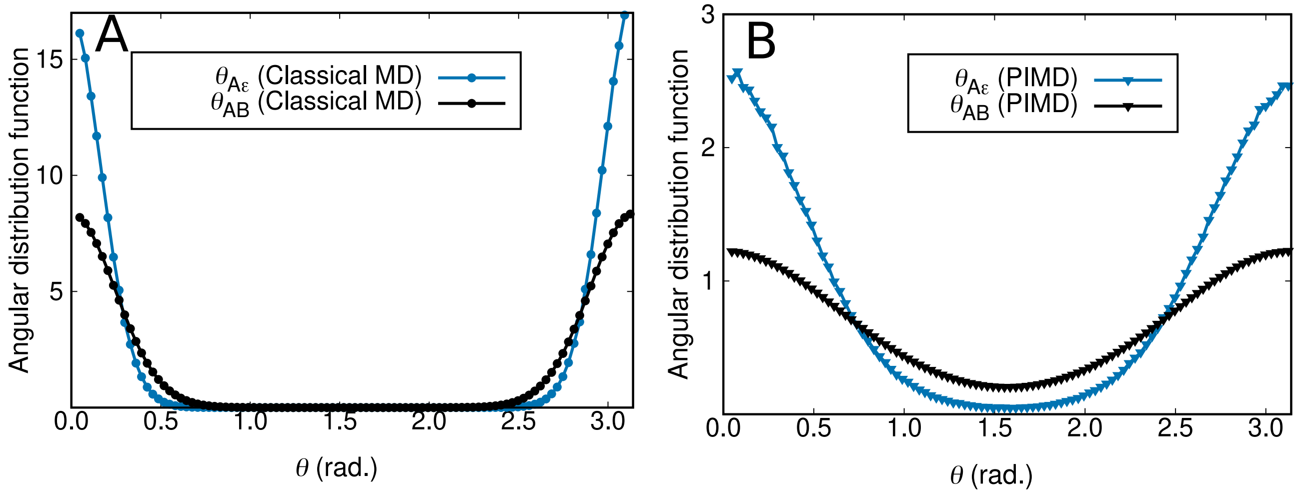

Fig. S6: Angular distribution functions of molecular bond axis of molecule A to the molecular bond axis of molecule B ($\theta_{\rm AB}$) and angular distribution functions of molecular bond axis of molecule A to the cavity polarization vector ($\theta_{\rm A\varepsilon}$) for $1,000$ H_2 molecules of a (A) classical MD simulation and (B) PIMD simulation are shown. Pair interaction potentials used for the MD simulation were obtained by training an ML model with the calculated energies from QED-CCSD-12-SD1 level of theory.

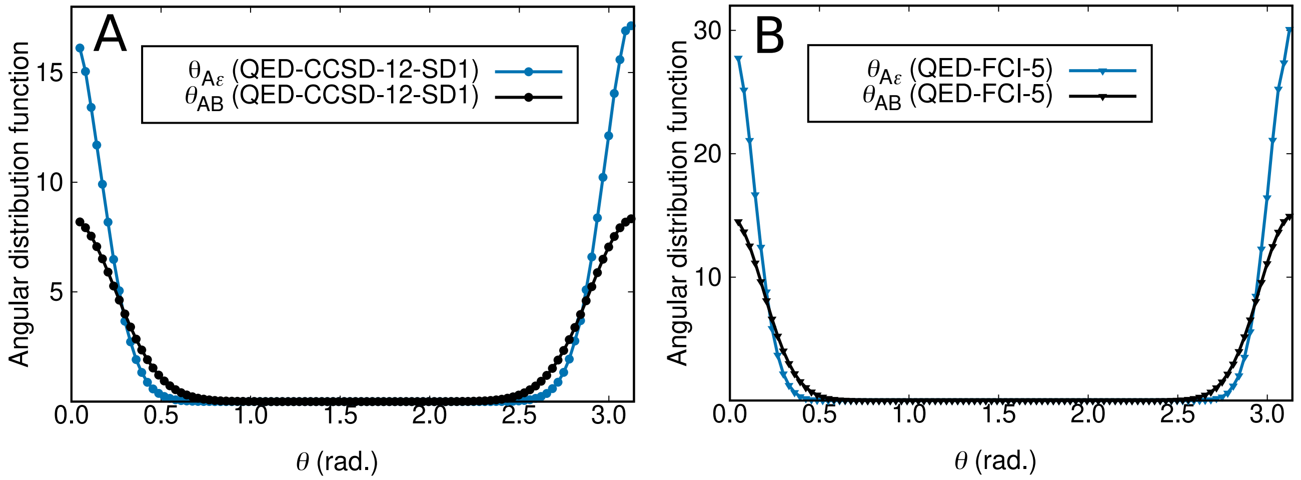

Fig. S7: Angular distribution functions of molecular bond axis of molecule A to the molecular bond axis of molecule B ($\theta_{\rm AB}$) and angular distribution functions of molecular bond axis of molecule A to the cavity polarization vector ($\theta_{\rm A\varepsilon}$) for $1,000$ H_2 molecules of a classical MD trajectory with the NN potentials obtained from training the ML model on (A) QED-CCSD-12-SD1 and (B) QED-FCI-5 data sets.

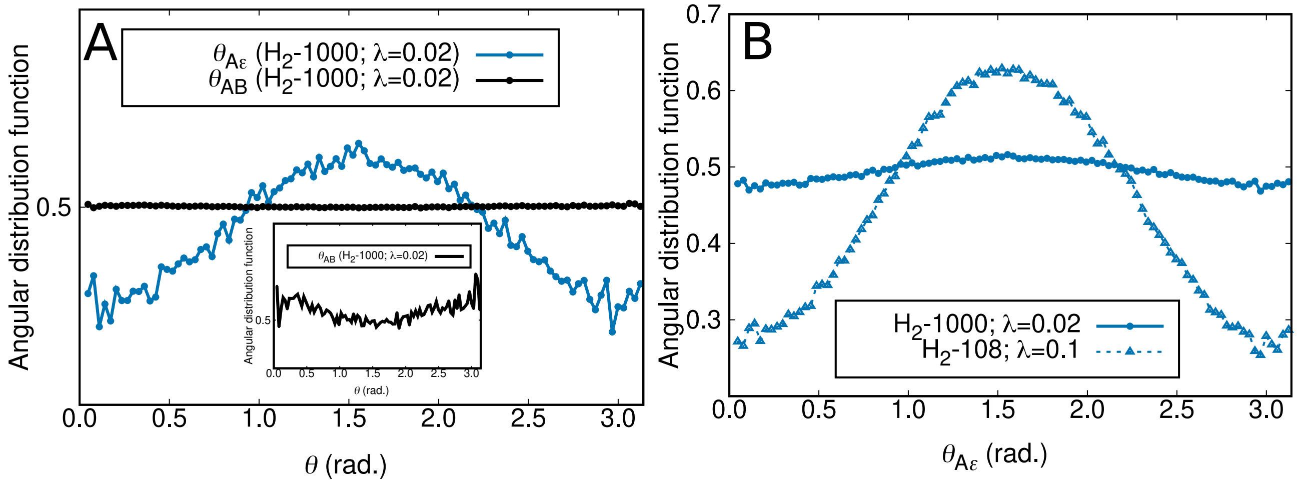

Fig. S8: Angular distribution functions of molecular bond axis of molecule A to the molecular bond axis of molecule B (θ_{AB}) and angular distribution functions of molecular bond axis of molecule A to the cavity polarization vector ($\theta_{\mathrm{A}\varepsilon}$) for $1,000$ H_2 molecules of a classical MD trajectory with the NNPs obtained from the training ML model on (A) QED-CCSD-12-SD1 and $\lambda = 0.02$ a.u. coupling constant are shown. A zoom-in figure of $\theta_{\mathrm{A}\varepsilon}$ is shown in the inset. (B) Angular distribution functions of molecular bond axis of molecule A to the cavity polarization vector ($\theta_{\mathrm{A}\varepsilon}$) of 108 molecules with $\lambda = 0.1$ a.u. (dashed line) and $1,000$ molecules with $\lambda = 0.02$ a.u. (solid line) are shown.

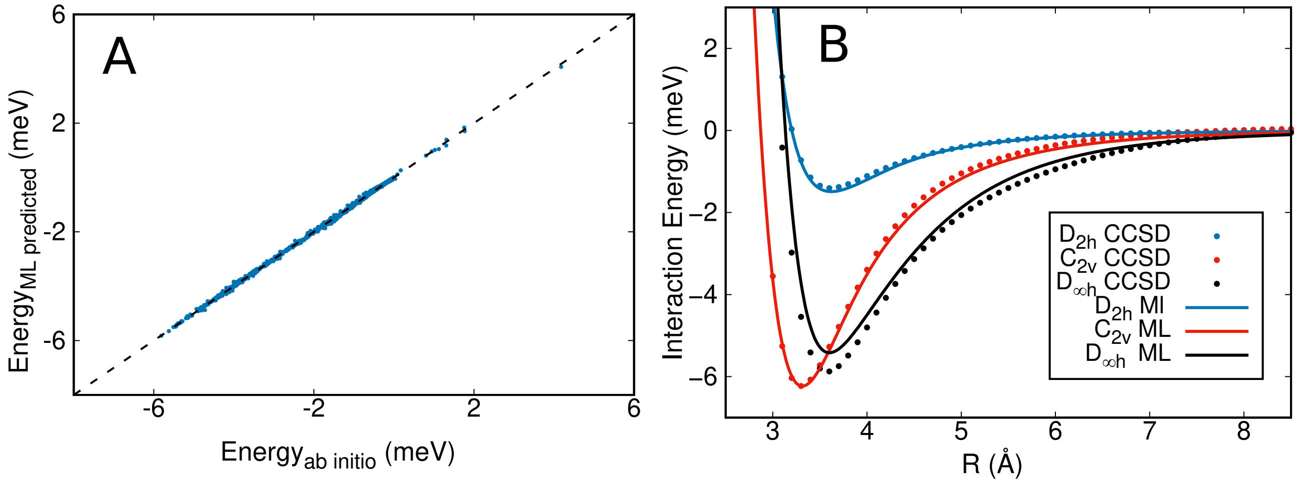

Fig. S9: (A) Pairwise interaction energies obtained from *ab initio*, CCSD calculation (without cavity) and ML predicted energies are plotted. (B) Scanned potential energy curve for D_{2h}, C_{2v} and $D_{\infty h}$ configuration of a pair of molecules using NNPs and from *ab initio* calculation are shown.

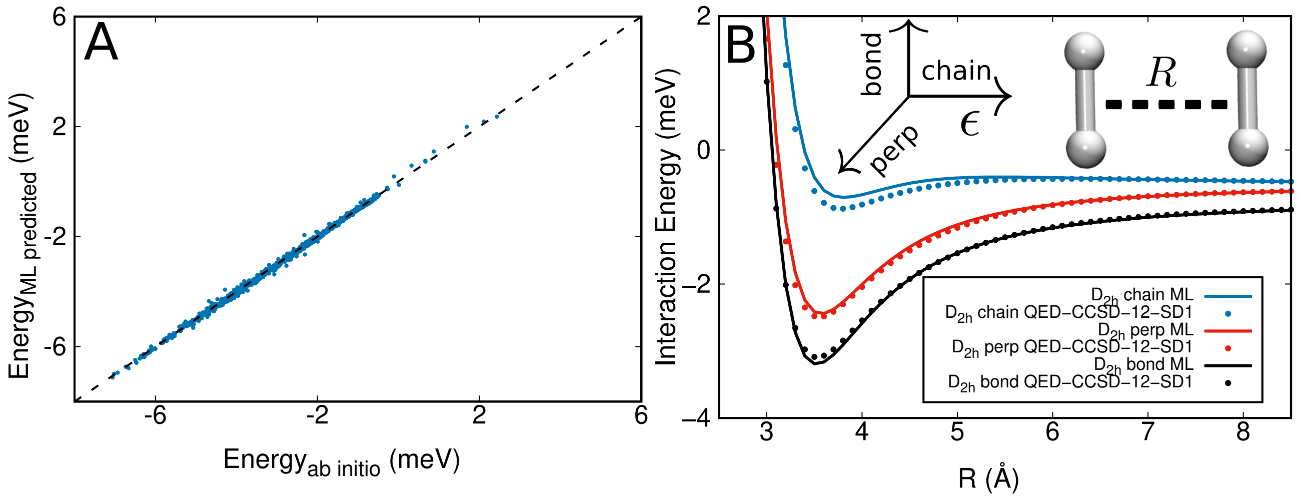

Fig. S10: (A) Pairwise interaction energies obtained from *ab initio*, QED-CCSD-12-SD1 calculation (with cavity) and ML predicted energies are plotted. (B) Scanned potential energy curve for D_{2h} configuration with three different direction of cavity polarization using NNPs and from *ab initio* calculation are shown.

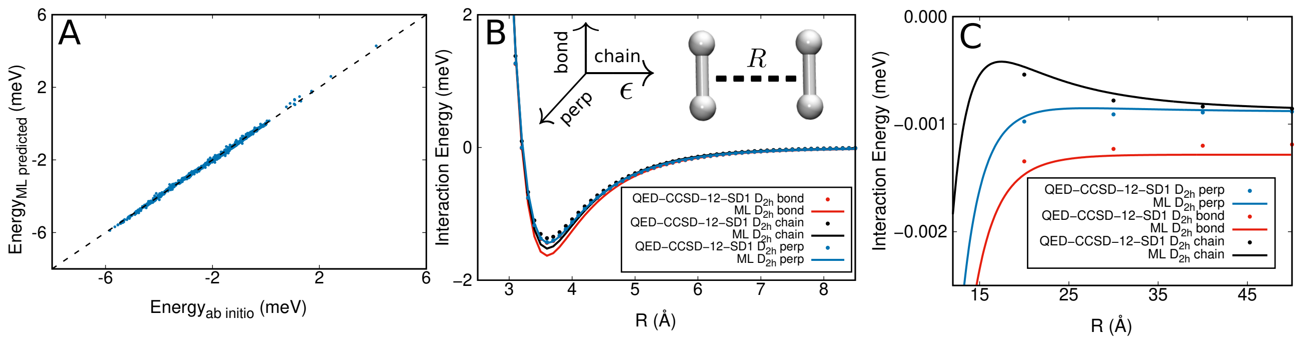

Fig. S11: (A) Pairwise interaction energies obtained from *ab initio*, QED-CCSD-12-SD1 calculation and ML predicted energies with $\lambda = 0.02$ a.u. are plotted. (B) Scanned potential energy curves for D_{2h} configuration of a pair of molecules using NNPs and from *ab initio* calculation are shown. Distance (R) between molecule A and molecule B over which potential energy is scanned is shown in the inset of the figure. (C) Scanned potential energy curves for D_{2h} configuration at the long range are shown. ML model can accurately distinguish different configurations at long distance.

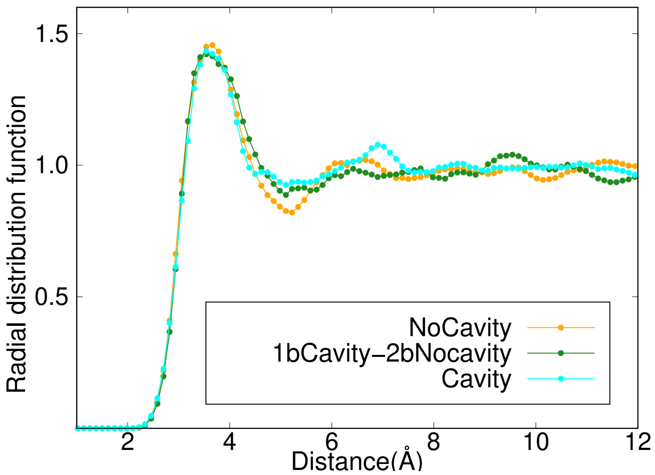

Fig. S12: Radial distribution function generated using PIMD trajectory with 1000 H_2 molecules using pair potential obtained through a ML training on *ab initio* calculation with QED-CCSD-12-SD1 and $\lambda = 0.1$ a.u.

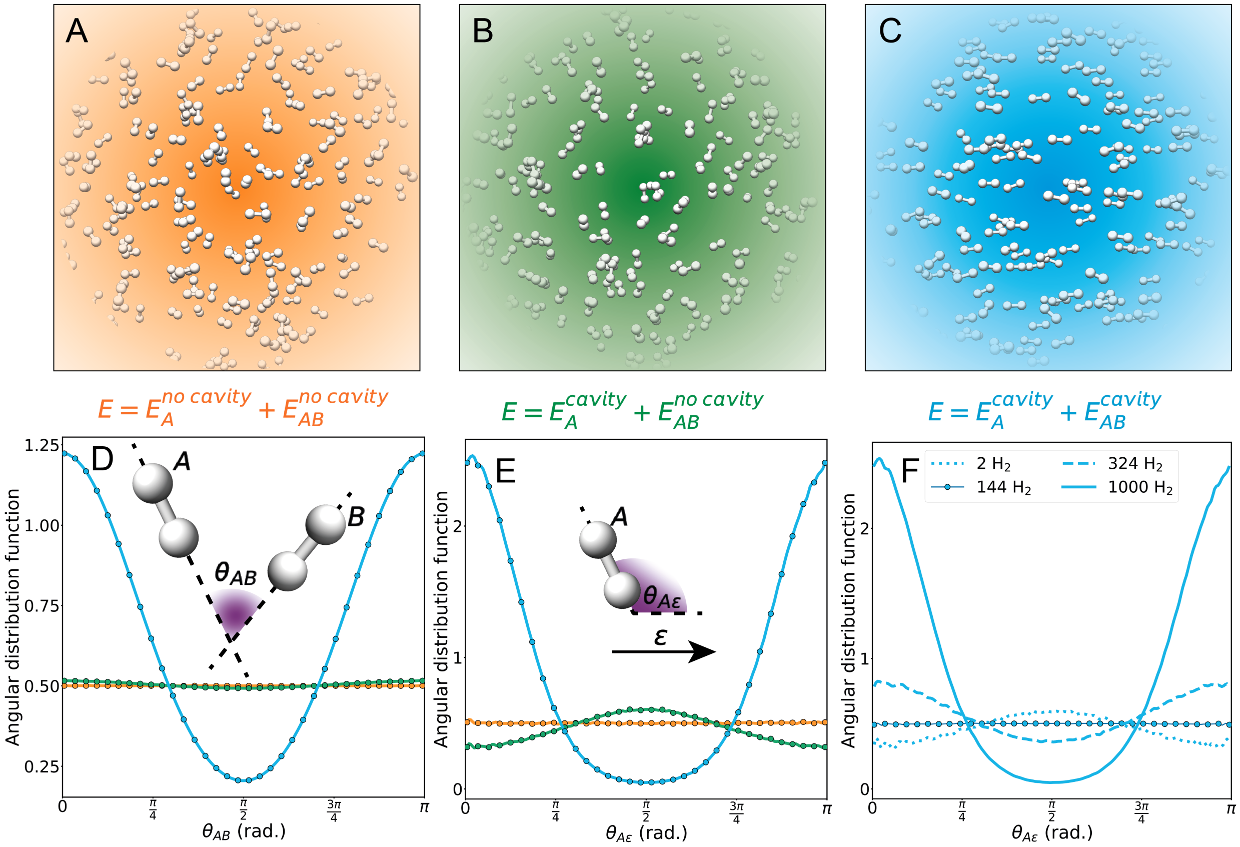

Fig. S13: (A-C) Snapshots taken at thermal equilibrium from the path integral molecular dynamic (PIMD) simulations of 1000 H_2 molecules in the case of (A) no cavity (orange), (B) cavity-modified one-body term but no cavity two-body term (green), and (C) cavity-modified one-body and two-body terms (blue). For these three cases, the (D) molecular bond axis of molecule A to molecular bond axis of molecule B (θ_{AB}) angular probability distribution function, $P(\theta_{AB})$ and (E) molecular bond axis to cavity polarization vector ($\theta_{A\varepsilon}$), angular probability distribution function, $P(\theta_{A\varepsilon})$, are shown. (F) molecular bond axis to cavity polarization vector ($\theta_{A\varepsilon}$), angular probability distribution function, $P(\theta_{A\varepsilon})$, are shown for four different simulations containing different numbers of H_2 molecules. All PIMD simulations shown in this figure were performed using neural networks trained with CCSD (no cavity) or QED-CCSD-12-SD1 with $\lambda = 0.1$ a.u. (cavity) calculated energies.

REFERENCES

[1] Haugland, T. S.; Schäfer, C.; Ronca, E.; Rubio, A.; Koch, H. Intermolecular Interactions in Optical Cavities: An *ab initio* QED Study. *J. Chem. Phys.* **2021**, *154*, 094113.

[2] White, A. F.; Gao, Y.; Minnich, A. J.; Chan, G. K. L. A Coupled Cluster Framework for Electrons and Phonons. *J. Chem. Phys.* **2020**, *153*, 224112.

[3] Haugland, T. S.; Ronca, E.; Kjønstad, E. F.; Rubio, A.; Koch, H. Coupled Cluster Theory for Molecular Polaritons: Changing Ground and Excited States. *Phys. Rev. X* **2020**, *10*, 041043.

[4] Eisenschitz, R.; London, F. Über das Verhältnis der van der Waalsschen Kräfte zu den homöopolaren Bindungskräften. *Zeitschrift für Phys.* **1930**, *60*, 491–527.

[5] London, F. Zur Theorie und Systematik der Molekularkräfte. *Zeitschrift für Phys.* **1930**, *63*, 245–279.

[6] London, F. The General Theory of Molecular Forces. *Trans. Faraday Soc.* **1937**, *33*, 8b–26.

[7] Thirunamachandran, T. Intermolecular Interactions in the Presence of an Intense Radiation Field. *Mol. Phys.* **1980**, *40*, 393–399.

[8] Milonni, P. W.; Smith, A. van der Waals Dispersion Forces in Electromagnetic Fields. *Phys. Rev. A* **1996**, *53*, 3484–3489.

[9] Sherkunov, Y. Casimir-Polder Interaction Between Two Atoms in Electromagnetic Fields. *J. Phys. Conf. Ser.* **2009**, *161*, 012041.

[10] Fiscelli, G.; Rizzuto, L.; Passante, R. Dispersion Interaction Between Two Hydrogen Atoms in a Static Electric Field. *Phys. Rev. Lett.* **2020**, *124*, 013604.

[11] Dahlke, E. E.; Truhlar, D. G. Electrostatically Embedded Many-Body Expansion for Large Systems, with Applications to Water Clusters. *J. Chem. Theory Comput.* **2007**, *3*, 46–53.

[12] Barron, J. T. Continuously Differentiable Exponential Linear Units. 2017; https://arxiv.org/abs/1704.07483.

[13] Kingma, D. P.; Ba, J. Adam: A Method for Stochastic Optimization. 2014; https://arxiv.org/abs/1412.6980.

[14] Paszke, A. et al. In *Advances in Neural Information Processing Systems 32*; Wallach, H., Larochelle, H., Beygelzimer, A., d'Alché-Buc, F., Fox, E., Garnett, R., Eds.; Curran Associates, Inc., 2019; pp 8024–8035.

[15] Bussi, G.; Parrinello, M. Accurate Sampling Using Langevin Dynamics. *Phys. Rev. E* **2007**, *75*, 056707.

[16] Ceriotti, M.; Bussi, G.; Parrinello, M. Nuclear Quantum Effects in Solids Using a Colored-Noise Thermostat. *Phys. Rev. Lett.* **2009**, *103*, 030603.

[17] Ceriotti, M.; Bussi, G.; Parrinello, M. Colored-Noise Thermostats à la Carte. *J. Chem. Theory Comput.* **2010**, *6*, 1170–1180.

[18] Ceriotti, M.; Manolopoulos, D. E.; Parrinello, M. Accelerating the Convergence of Path Integral Dynamics with a Generalized Langevin Equation. *J. Chem. Phys.* **2011**, *134*, 084104.

[19] Ceriotti, M.; Parrinello, M.; Markland, T. E.; Manolopoulos, D. E. Efficient Stochastic Thermostatting of Path Integral Molecular Dynamics. *J. Chem. Phys.* **2010**, *133*, 124104.

[20] Ceriotti, M.; More, J.; Manolopoulos, D. E. i-PI: A Python Interface for *ab initio* Path Integral Molecular Dynamics Simulations. *Comput. Phys. Commun.* **2014**, *185*, 1019–1026.
